# The association between triglyceride glucose index and arthritis: a population-based study

**DOI:** 10.1186/s12944-023-01899-9

**Published:** 2023-08-22

**Authors:** Yuxin Yan, Liyu Zhou, Rui La, Ming Jiang, Dinghua Jiang, Lixin Huang, Wu Xu, Qian Wu

**Affiliations:** Department of Orthopedic Surgery, Orthopedic Institute, The First Affiliated Hospital, Suzhou Medical College, Soochow University, Suzhou, Jiangsu China

**Keywords:** Arthritis, Triglyceride glucose index, National Health and Nutrition Examination Survey, Cross-sectional study, Insulin resistance

## Abstract

**Objectives:**

Insulin resistance is a well-established contributor to inflammation; however, the specific association between the triglyceride glucose (TyG) index, a biomarker reflecting insulin resistance, and arthritis remains unexplored. As a result, the main aim of this study was to examine the correlation between the TyG index and arthritis.

**Methods:**

This observational study used data from the National Health and Nutrition Examination Survey (NHANES), which was conducted between 2007 and 2018. To investigate the relationship between the TyG index and arthritis, various statistical analyses were employed, including weighted multivariable logistic regression analysis, subgroup analysis, curve fit analysis, and threshold effect analysis.

**Results:**

In total, 14,817 patients were enrolled in the trial, with 4,191 individuals (28.29%) diagnosed with arthritis. An increased risk of arthritis was found to be significantly correlated with higher TyG index values (odds ratio OR = 1.15, 95% confidence interval CI: 1.07–1.23), according to the results of multivariable logistic regression analysis after full adjustment. Subgroup analysis and interaction tests further indicated that the TyG index exhibited an additive effect when combined with other established risk factors, including age (OR = 1.29; 95% CI: 1.17–1.41), body mass index (BMI) (OR = 1.43; 95% CI: 1.24–1.69), and diabetes (OR = 1.20; 95% CI: 1.11–1.31). Additionally, curve fit analysis and threshold effect analysis demonstrated a nonlinear relationship with a breakpoint identified at 8.08 µmol/L.

**Conclusion:**

The TyG index was positively correlated with arthritis in adults under 60 years of age in the United States who had normal weight and no diabetes. Further large-scale prospective studies are warranted for a comprehensive analysis of the role of the TyG index in arthritis.

**Supplementary Information:**

The online version contains supplementary material available at 10.1186/s12944-023-01899-9.

## Introduction

Arthritis is a complex and widespread medical condition characterized by inflammation and stiffness in one or more joints of the body [1]. By 2040, 78.4 million adults in the United States will be diagnosed with arthritis, approximately 25.9% of all adults [2]. It is characterized by inflammation and stiffness in one or more joints of the body, with the most prevalent forms being osteoarthritis, rheumatoid arthritis, and psoriatic arthritis [3; 4]. Although there are differences in the pathological process of different types of arthritis, arthritis is related to inflammation [5]. All of them have the potential to significantly lower patients’ quality of life by causing joint discomfort, functional limitations, and diminished mobility [6; 7]. According to the World Health Organization (WHO), arthritis has emerged as a prevalent cause of disability in the United States. Obviously, the impact of arthritis extends beyond individual suffering, as it imposes substantial economic and healthcare burdens on societies [8; 9]. Given the rising incidence of arthritis and its associated medical costs, early screening of high-risk groups based on risk factors and the development of effective approaches to management are vital for both individuals and society.

The TyG index is an emerging marker that has gained recognition for its utility in assessing insulin resistance and metabolic dysfunction [10]. In the past five years, the TyG index has garnered significant attention as a simple and cost-effective method for assessing insulin resistance, especially when compared to traditional complex techniques such as the euglycemic clamp or the Homeostatic Model Assessment of Insulin Resistance (HOMA-IR) [11; 12]. The appeal of the TyG index lies in its utilization of readily available clinical measurements, including fasting triglyceride and glucose levels, which are routinely obtained in clinical practice. The TyG index has been demonstrated to be a surrogate marker for identifying individuals at risk for metabolic conditions such as type 2 diabetes, nonalcoholic fatty liver disease, and coronary artery disease in several investigations [13; 14; 15]. Its simplicity and potential clinical relevance make the TyG index a potentially promising tool for assessing insulin resistance and its implications in various metabolism-related diseases.

Insulin resistance is linked to chronic low-level inflammation, and inflammation is closely linked to joint disease [5; 16]. As a result, studies related to insulin and arthritis have gained importance in recent years. According to the study conducted by Hamada et al. [17], insulin has been found to play a protective and anti-inflammatory role within the synovium. However, the presence of insulin resistance can hinder this beneficial effect, thereby potentially contributing to joint damage. Araújo et al. [18] found that insulin resistance can affect bone mass and reduce bone density. Furthermore, insulin has been shown to have direct effects on cartilage metabolism, promoting the synthesis of inflammatory mediators and degrading enzymes that can contribute to cartilage degradation and joint inflammation [19]. However, previous studies focused on cellular and animal studies, and there is a scarcity of large-scale population-based investigations focusing on insulin resistance and arthritis risk.

Therefore, the goal of this study was to determine the association between the TyG index and arthritis by conducting a comprehensive cross-sectional analysis utilizing the extensive dataset of the NHANES.

## Methods

### Survey description

NHANES is a large-scale cross-sectional survey conducted in the United States aimed at collecting data on the health and nutritional status of the general population. NHANES utilizes a stratified multistage random sampling approach to ensure representative coverage [20; 21]. The National Center for Health Statistics study ethical review board approved NHANES, and each participant signed written agreement forms to give their informed consent [22]. The NHANES datasets, along with the accompanying documentation and protocols, can be freely obtained from the website.

### Study population

For this analysis, data from six cycles of two years, encompassing the years 2007 to 2018, were collected. All measurements and tests were conducted at mobile testing facilities set up on-site, ensuring standardized procedures and data collection protocols.

Our study applied specific exclusion criteria to ensure the validity and reliability of our findings. The following criteria were used for study exclusions: (1) individuals below 20 years of age, as our focus was on adult populations; (2) pregnant women, as their metabolic profiles can be altered during pregnancy; (3) individuals with missing arthritis data, as their inclusion could introduce bias in our analysis; and (4) individuals with missing data on triglyceride and glucose levels, as these variables were essential for calculating the TyG index. Participants with missing covariate data were excluded from the study.

### Assessment of the TyG index

The formula for calculating the TyG index is as follows: TyG = Ln [fasting triglycerides (mg/dL) x fasting glucose (mg/dL)/2] [23]. This equation represents a logarithmic transformation of the product obtained by multiplying the fasting triglyceride and glucose levels, divided by 2. In our analysis, we treated the TyG index as a continuous variable and subsequently categorized participants into tertiles based on their TyG index value for further investigation. It is important to note that the TyG index was considered an exposure variable within the framework of our study design.

### Assessment of the diagnosis of arthritis

Arthritis diagnosis was ascertained through a self-report questionnaire (MCQ160a), which included the following question: “Has a healthcare professional ever informed you that you have arthritis?“ Participants were asked to choose between two response options: “Yes” or “No.” [24].

### Assessment of covariables of interest

The study incorporated various covariates to account for potential confounding factors. These covariates included age, sex, race, body mass index (BMI), hypertension, diabetes, poverty-to-income ratio (PIR), marital status, education level, moderate activity, and vigorous activity. Age was categorized into two groups: “Below 60” and “Over 60,“ enabling a comparison of the effects between different age ranges. Two categories of marital status were identified: “Married or with partner” and “Single,“ allowing for an examination of potential differences based on relationship status. BMI was divided into three groups using cutoffs of 25 and 30: less than 25 is normal weight, greater than or equal to 30 is obese, and between the cutoffs is overweight. The PIR, which was divided into three levels, “1,“ “1–5,“ and “5,“ was used as a proxy indicator of socioeconomic status (SES).

### Statistical analyses

All statistical analyses were performed using R software (version 4.1.3) and EmpowerStats (version: 2.0), and the significance level was set at *P* < 0.05. Participants with missing covariate data were excluded from the study. The triglyceride glucose index was divided into tertiles, with the lowest tertile (T1) serving as the reference group. Continuous variables are presented as medians ± interquartile ranges according to their distribution status, whereas categorical variables are expressed as frequencies and percentages. The chi-squared test or Kruskal‒Wallis H test was employed for different TyG index tertile groups. Multivariate logistic regression analysis was conducted to investigate the relationship between the triglyceride glucose index and arthritis. Model 1 was unadjusted, while Model 2 was adjusted for sex age, and race. Finally, Model 3, which was our core model, was adjusted for the variables in Model 2 as well as history of education level, poverty-to-income ratio, marital status, body mass index, hypertension, diabetes, moderate activity, and vigorous activity. The same statistical methods presented above were also applied to the age, BMI, and diabetes subgroups, allowing for subgroup analyses to examine potential variations within these specific populations. To better evaluate the nonlinear relationship between the TyG index and arthritis, our study incorporated smooth curve fitting and threshold effect analysis. These statistical techniques enabled a more comprehensive exploration of the potential nonlinearity between the TyG index and the risk of arthritis.

## Results

**Fig. 1:**
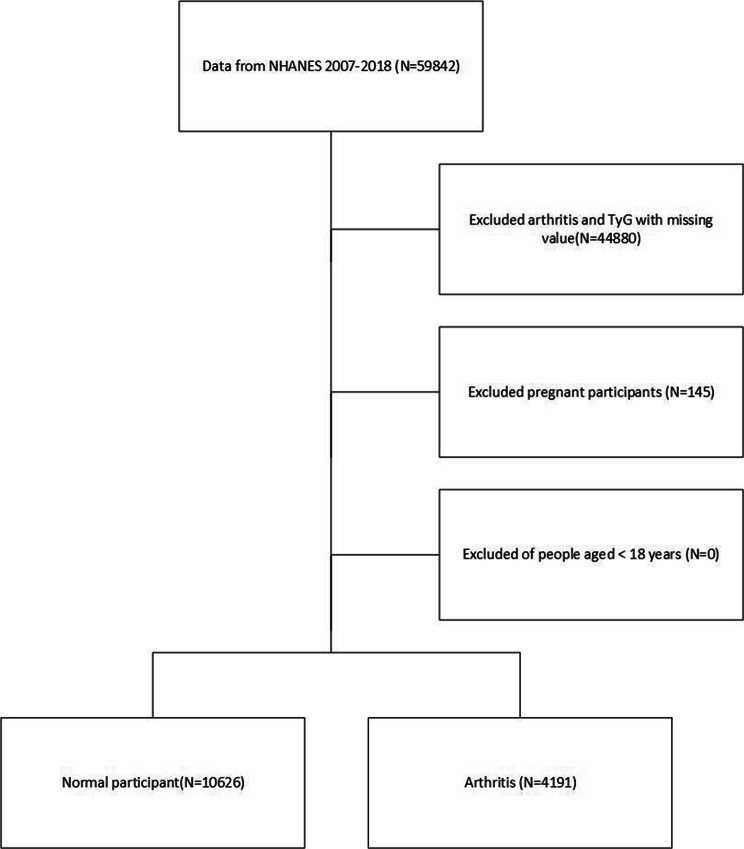
Flowchart of participant selection from NHANES 2007–2018

After applying exclusion criteria, our final study cohort comprised a total of 14,817 participants. For a more comprehensive understanding of our study design, sampling methods, and specific exclusion criteria, we refer readers to Fig. 1, which provides additional details on these aspects.

### Baseline characteristics of participants

Table 1 presents the baseline characteristics of the participants in the study. A total of 14,817 participants met the selection criteria and were included, with an overall incidence of arthritis in this cohort of 28.29%. Among the participants, 65.33% were below 60 years of age, while 34.67% were aged 60 years or older. In terms of sex distribution, 48.82% were males, and 51.18% were females. Regarding ethnicity, 15.26% were of Mexican American descent, 10.95% were from other Hispanic backgrounds, 41.49% were non-Hispanic whites, 20.02% were non-Hispanic blacks, and 12.28% belonged to other racial groups. The median ± interquartile range concentrations of triglycerides, glucose, and the TyG index were 101.00 ± 79, 101.00 ± 18, and 8.57 ± 0.87, respectively. The participants were classified into tertiles based on their triglyceride glucose index values, with T1 representing values ranging from 5.64 to 8.30, T2 ranging from 8.30 to 8.85, and T3 ranging from 8.85 to 12.84. A significant progressive gain in the prevalence of arthritis was observed as the participants’ triglyceride index values increased (T1: 21.67%, T2: 29.14%, T3: 34.04%, *P* < 0.001).

**Table 1:** Baseline characteristics of the study population according to the TyG index in NHANES 2007–2018

	Overall (5.64–12.84)	Tertile 1 (5.64–8.30)	Tertile 2 (8.30–8.85)	Tertile 3 (8.85–12.84)	*P* value
Age (%)					< 0.001
Below60	65.33	74.66	62.70	58.64	
Over60	34.67	25.34	37.30	41.36	
Sex (%)					< 0.001
Male	48.82	40.75	49.95	55.74	
Female	51.18	59.25	50.05	44.26	
Race (%)					< 0.001
Mexican American	15.26	10.53	15.52	19.72	
Other Hispanic	10.95	8.75	11.48	12.63	
Non-Hispanic White	41.49	38.24	42.36	43.86	
Non-Hispanic Black	20.02	29.31	18.58	12.19	
Other Races	12.28	13.17	12.06	11.60	
Marital status (%)					< 0.001
Married or with partner	59.70	56.31	60.38	62.42	
Single	40.30	43.69	39.62	37.58	
Education level (%)					< 0.001
Less than high school	25.22	19.14	25.25	31.28	
High school or GED	22.59	20.88	23.69	23.21	
Above high school	52.19	59.98	51.06	45.51	
PIR (%)					0.004
< 1	21.76	20.92	21.46	22.91	
1–5	61.38	60.75	60.72	62.67	
>=5	16.86	18.33	17.82	14.41	
BMI category (%)					< 0.001
Normal weight	429.07	45.00	27.59	14.53	
Overweight	33.04	29.34	35.76	34.05	
Obese	37.89	25.66	36.65	51.43	
Vigorous activity (%)					0.307
Yes	19.41	20.06	19.31	18.85	
No	80.59	79.94	80.69	81.15	
Moderate activity (%)					0.014
Yes	36.69	38.22	36.42	35.42	
No	63.31	61.78	63.58	64.58	
Hypertension (%)					< 0.001
Yes	37.43	27.00	36.47	48.84	
No	62.57	73.00	63.53	51.16	
Diabetes					< 0.001
Yes	13.99	4.83	10.19	27.10	
No	12,426	95.17	89.81	72.90	
Triglyceride (mg/dl)	101.00 ± 79	61.00 ± 23	103.00 ± 28	177.00 ± 82	< 0.001
Glucose (mg/dl)	101.00 ± 18	95.00 ± 12	101.00 ± 14	111.00 ± 35	< 0.001
TyG index	8.57 ± 0.87	7.98 ± 0.41	8.57 ± 0.27	9.23 ± 0.54	< 0.001
Arthritis (%)					< 0.001
Yes	28.29	21.67	29.14	34.04	
No	71.71	78.33	70.86	65.96	

The characteristics of participants with arthritis and no arthritis are illustrated in Table 2. Compared with the individuals without arthritis, participants with arthritis were older than 60 years of age. A higher percentage of women had arthritis than men. There was a higher percentage of non-Hispanic white participants with arthritis than people without arthritis. Participants with arthritis had a higher prevalence of education level, obesity and hypertension. The TyG index was higher among arthritis participants than participants without arthritis.

**Table 2:** Baseline characteristics of the arthritis group versus the nonarthritis group

	Arthritis	Non-Arthritis	*P* value
Age (%)			< 0.001
Below60	38.39	75.96	
Over60	61.61	24.04	
Sex (%)			< 0.001
Male	40.94	51.92	
Female	59.06	48.08	
Race (%)			< 0.001
Mexican American	11.26	16.84	
Other Hispanic	9.47	11.54	
Non-Hispanic White	51.40	37.58	
Non-Hispanic Black	20.76	19.73	
Other Races	7.11	14.31	
Marital status (%)			0.021
Married or with partner	58.22	60.29	
Single	41.78	39.71	
Education level (%)			< 0.001
Less than high school	28.25	24.02	
High school or GED	23.72	22.15	
Above high school	48.03	53.83	
PIR (%)			0.153
< 1	22.04	21.65	
1–5	62.10	61.09	
>=5	15.86	17.25	
BMI (%)			< 0.001
Normal weight	21.18	32.16	
Overweight	30.90	33.88	
Obese	47.92	33.96	
Vigorous activity (%)			< 0.001
Yes	15.59	20.91	
No	84.41	79.09	
Moderate activity (%)			< 0.001
Yes	33.46	37.96	
No	66.54	62.04	
Hypertension (%)			< 0.001
Yes	60.32	28.40	
No	39.68	71.60	
Diabetes			< 0.001
Yes	23.90	10.15	
No	76.10	89.85	
Triglyceride (mg/dL)	110.00 ± 81	98.00 ± 78	< 0.001
Glucose (mg/dL)	105.00 ± 23	100.00 ± 17	< 0.001
TyG index	8.70 ± 0.84	8.52 ± 0.88	< 0.001

### Associations between the TyG index and arthritis

Table 3 displays the results of the multivariate logistic regression study looking at the association between the TyG index and arthritis. In the unadjusted model, a highly significant positive correlation was observed between the TyG index and arthritis (OR = 1.47; 95% CI: 1.39–1.54; *P* < 0.0001). Upon adjusting for sex, age, and race variables in model 2, this significant positive association remained evident (OR = 1.46; 95% CI: 1.38–1.55; *P* < 0.0001). Even after accounting for all covariates in model 3, the relationship between the TyG index and arthritis remained significant and positive (OR = 1.15; 95% CI: 1.07–1.23; *P* = 0.0002). Additionally, when fully adjusting for potential interfering factors, the odds ratios (ORs) with corresponding confidence intervals (CIs) indicated a significant positive association in T2 and T3 compared to T1. Specifically, the ORs were 1.15 (95% CI: 1.03–1.28, P for trend < 0.0001) and 1.19 (95% CI: 1.05–1.34, *P* for trend = 0.0061) for T2 and T3, respectively, when compared to T1.

**Table 3:** Odds ratios and 95% confidence intervals for arthritis according to the TyG index

	OR (95% CI), *P* value		
	Model 1^1^	Model 2^2^	Model 3^3^
	n = 14,817	n = 14,817	n = 12,888
Arthritis			
TyG	1.47 (1.39, 1.54) < 0.0001	1.46 (1.38, 1.55) < 0.0001	1.15 (1.07, 1.23) 0.0002
Categories			
Tertile 1	Reference	Reference	Reference
Tertile 2	1.49 (1.36, 1.63) < 0.0001	1.36 (1.23, 1.50) < 0.0001	1.15 (1.03, 1.28) 0.0162
Tertile 3	1.86 (1.70, 2.04) < 0.0001	1.73 (1.57, 1.92) < 0.0001	1.19 (1.05, 1.34) 0.0048
P for trend	< 0.0001	< 0.0001	0.0061

In the sensitivity analysis, TyG was converted from a continuous variable to a categorical variable (tertiles)

OR: odds ratio; 95% Cl: 95% confidence interval

^1^Model 1: No covariates were adjusted

^2^Model 2: Adjusted for sex, age, and race

^3^Model 3: Adjusted for sex, age, race, education level, PIR, marital status, BMI, hypertension, diabetes, moderate activity and vigorous activity

### Subgroup analyses

Subgroup analyses were performed to estimate the associations between the TyG index and arthritis, and the results are presented in Table 4. Interestingly, some associations remained consistent across different subgroups, including sex, hypertension, vigorous activity, and moderate activity. However, notable interactions were observed between age, BMI, diabetes, and the associations with the TyG index. Subgroup analyses stratified by age, BMI, and diabetes revealed intriguing findings. The positive association between the TyG index and arthritis was more pronounced in participants below 60 years of age (OR = 1.29; 95% CI: 1.17–1.41), those without diabetes (OR = 1.20; 95% CI: 1.11–1.31), and individuals with normal weight (OR = 1.43; 95% CI: 1.24–1.69).

**Table 4:** Subgroups analysis for the associations between the TyG index and arthritis

Variables	OR(95%CI)	*P*	*P* for interaction
**Age (years)**			< 0.0001
Below60	1.29 (1.17, 1.41)	< 0.0001	
Over60	0.91 (0.81, 1.01)	0.0846	
**Sex**			0.4923
male	1.11 (1.00, 1.23)	0.0418	
female	1.17 (1.06, 1.30)	0.0022	
**BMI**			0.0037
Normal weight	1.43 (1.24, 1.69)	< 0.0001	
Overweight	1.11 (0.98, 1.25)	0.1007	
Obese	1.06 (0.95, 1.18)	0.2871	
**Hypertension**			0.0633
Yes	1.07 (0.97, 1.18)	0.1749	
No	1.22 (1.11, 1.35)	< 0.0001	
**Diabetes**			0.0012
Yes	0.92 (0.80, 1.06)	0.2475	
No	1.20 (1.11, 1.31)	< 0.0001	
**Vigorous activity**			0.1473
Yes	1.27 (1.08, 1.48)	0.0030	
No	1.12 (1.03, 1.21)	0.0061	
**Moderate activity**			0.1891
Yes	1.21 (1.08, 1.36)	0.0009	
No	1.11 (1.01, 1.21)	0.0258	

### Nonlinear association between the TyG index and arthritis

**Fig. 2:**
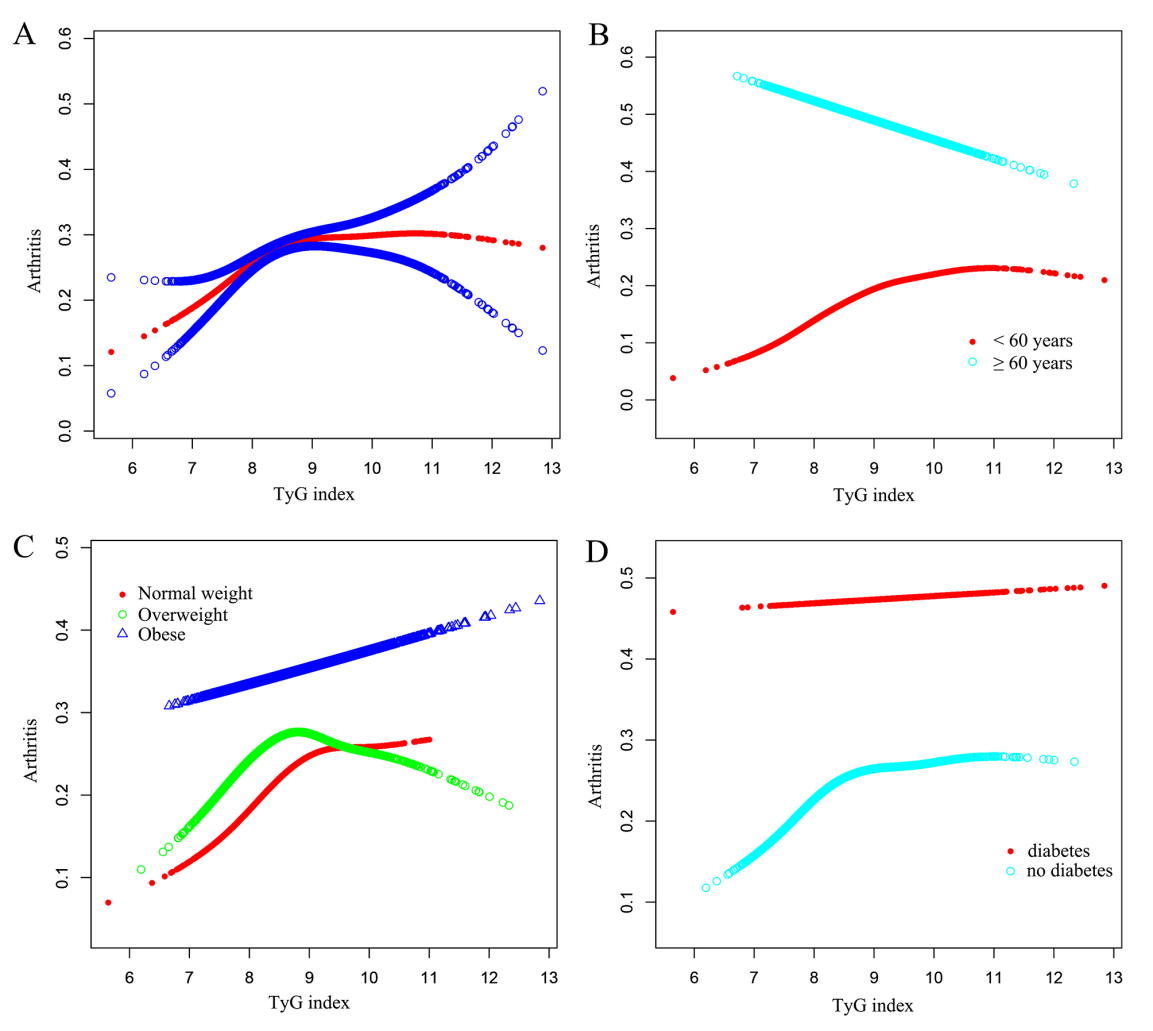
The association between the TyG index and arthritis. **(A)** The solid red line represents the smooth curve fit between variables. Blue bands represent the 95% confidence bands derived from the fit. **(B)** Stratified by age. **(C)** Stratified by BMI. **(D)** Stratified by diabetes

The adjusted smoothing curve depicted in Fig. 2 demonstrates a saturation effect value relationship between the TyG index and arthritis. Utilizing a two-segment linear regression model, we calculated the turning points to be 8.08 µmol/L for the TyG index. When the TyG index was below 8.08 µmol/L, we observed a significantly positive association with arthritis, with an OR value of 2.01 (95% CI: 1.46–2.75; *P* < 0.0001). However, once the TyG index reached 8.08 µmol/L, the OR value displayed a slow increase, eventually reaching a saturation point of 1.05 (95% CI: 0.97–1.15; *P* = 0.2235). Furthermore, we identified distinct inflection points and saturation effect values in the TyG index among individuals aged below 60 years old, without diabetes and with normal weight, with respective inflection points of 8.09, 8.07, and 9.06, as presented in Table 5.

**Table 5:** Threshold effect analysis of triglyceride glucose index and arthritis using the two-segment piecewise linear regression model

Arthritis	Adjusted β (95% CI) *P* value
TyG index	
Inflection point	8.08
TyG index < Inflection point	2.01 (1.46, 2.75) < 0.0001
TyG index > Inflection point	1.05 (0.97, 1.15) 0.2235
Log-likelihood ratio	< 0.001
**below 60 years**	
Inflection point	8.09
TyG index < Inflection point	2.80 (1.84, 4.25) < 0.0001
TyG index > Inflection point	1.13 (1.01, 1.27) 0.0368
Log-likelihood ratio	< 0.001
**no diabetes**	
Inflection point	8.07
TyG index < Inflection point	2.06 (1.46, 2.90) < 0.0001
TyG index > Inflection point	1.09 (0.98, 1.21) 0.1083
Log-likelihood ratio	0.001
**normal weight**	
Inflection point	9.06
TyG index < Inflection point	1.65 (1.34, 2.02) < 0.0001
TyG index > Inflection point	0.82 (0.49, 1.39) 0.4677
Log-likelihood ratio	0.025

Adjusted for sex, age, race, education level, PIR, marital status, BMI, hypertension, diabetes, moderate activity and vigorous activity.

## Discussion

In our analysis, we observed a robust correlation between the TyG index and arthritis, which persisted even after adjusting for relevant confounding variables in the fully adjusted model. Subgroup analysis and interaction tests showed that the TyG index may be particularly relevant in identifying individuals with arthritis who are below 60 years of age, have a BMI below 25.0, and do not have diabetes or hypertension. These findings suggest that the impact of the TyG index on arthritis may be influenced by age, BMI, and diabetes status, highlighting the importance of considering these factors when assessing the relationship between the TyG index and arthritis. Curve fit analysis and threshold effect analysis showed a nonlinear relationship with a breakpoint of 8.08 between the TyG index and arthritis. Focusing on the TyG index of patients is a simple but effective tool for the epidemiological study of arthritis.

Biomarkers focusing on arthritis have been progressively reported by scholars over the years as the NHANES database has been extensively studied. In a study conducted by Liu et al. [25], it was shown that the systemic immune-inflammation index serves as a novel and convenient marker of inflammation, suggesting its potential importance in assessing arthritis. Similarly, Xiang et al. [26] found that the dietary inflammation index has an additive effect alongside other risk factors, thereby increasing the likelihood of developing arthritis. Moreover, Chen et al. [27] revealed a significant association between sensitivity to thyroid hormone indices and the prevalence of osteoarthritis, highlighting the intricate correlation between the thyroid system and chondrogenic differentiation. These findings collectively underscore the significance of incorporating various markers in the assessment of arthritis. However, no studies have investigated the correlation between the TyG index, a novel and easy measure of insulin resistance and systemic inflammation, and arthritis.

To the best of our knowledge, this is the first cross-sectional study to investigate the relationship between the TyG index and arthritis. Prior investigations have examined the association between the TyG index and diverse diseases, employing a range of epidemiological methods and focusing on various target populations. According to Shi et al. [28], their study indicates a significant association between TyG index values and the presence of depressive symptoms among adults in the United States. Zheng et al. [29] reported a significant association between a higher TyG index and an increased risk of nephrolithiasis and its recurrence. Their findings suggest that early intervention and management of insulin resistance may potentially improve or alleviate the occurrence and recurrence of kidney stones. Ahn et al. [30] discovered a significant positive association between the TyG index and a low skeletal muscle mass index in Korean participants. According to the findings of Guo et al. [31], an elevated TyG index was associated with a higher risk of long-term complications following percutaneous coronary intervention, such as repeat revascularization and in-stent restenosis. The results presented by Guo et al. highlight the clinical relevance of the TyG index in identifying individuals at higher risk for adverse outcomes after percutaneous coronary intervention and emphasize its potential as a valuable tool in risk assessment and patient management within the context of chronic coronary syndrome. Ren et al. [32] suggested a potential association between the TyG index and the severity and morbidity of COVID-19, indicating that the TyG index could serve as a valuable marker for identifying poor outcomes in patients with COVID-19. Yilmaz et al. [33] conducted a cross-sectional study that revealed a statistically significant elevation of the TyG index in patients with erectile dysfunction (ED) compared to the non-ED group. These findings suggest that the TyG index may hold potential as a diagnostic and monitoring tool for ED. Obviously, there is increasing evidence that the TyG index is not only applicable to diabetics but may also be relevant to other diseases.

The underlying mechanism driving the association between the TyG index and arthritis remains unclear. The observed association suggests potential underlying mechanisms that link insulin resistance, metabolic dysfunction, and chronic inflammation to the pathogenesis of arthritis. The interaction between insulin resistance and inflammation is likely to be facilitated by several mechanisms. On the one hand, arthritis is known to cause the upregulation of proinflammatory cytokines, such as tumor necrosis factor (TNF) and interleukin (IL), which are closely associated with insulin resistance. Elevated systemic levels of TNF-α have been associated with the activation of key signaling proteins, including IKK, p38 MAPK, JNK, and PKC. When activated, these proteins directly target specific serine residues on the insulin receptor substrate (IRS) protein, disrupting its normal functioning. This disruption impedes the process of tyrosine phosphorylation, resulting in impaired insulin signaling and subsequent insulin resistance [34]. TNF-α also upregulates the expression of protein tyrosine phosphatase 1B (PTP1B), which acts by dephosphorylating phosphoserine residues present in insulin receptor and IRS proteins [35]. Through this mechanism, TNF-α-mediated induction of PTP1B disrupts the normal phosphorylation events required for optimal insulin receptor and IRS signaling, ultimately impairing insulin sensitivity and downstream metabolic responses. The upregulation of IL-6 can stimulate the JAK-STAT signaling pathways, resulting in an increase in the expression of suppressor of cytokine signaling 1 (SOCS1) and SOCS3 proteins. These proteins play a role in downregulating the function of the insulin receptor through mechanisms such as sterically blocking its interaction with IRS proteins or modifying kinase activity [36; 37; 38; 39]. Additionally, IL-6 can induce the expression of the TLR-4 gene by activating STAT3. When combined with IL-1β, IL-6 can enhance the activation of STAT3 and NF-κB in hepatocytes, leading to inflammation [40]. Furthermore, IL-1β activates p38 MAPK via the IL-1β receptor, which in turn hinders insulin signaling by inducing serine phosphorylation on IRS1/2 [41].

On the other hand, insulin resistance contributes to oxidative stress and the generation of reactive oxygen species, which are important in the progression of arthritis [42]. Elevated levels of reactive oxygen species (ROS) can have detrimental effects, encompassing not only oxidative damage but also disturbances in redox-regulated cell signaling pathways, such as Akt and MAP kinase signaling. Mitochondrial dysfunction, often observed with aging, plays a role in this process, as it leads to increased generation of ROS within the mitochondria. Age-related changes, including reduced activity of mitochondrial superoxide dismutase, contribute to the augmented production of ROS in chondrocytes, which exacerbates the oxidative stress associated with aging [43]. Consequently, the disruption of redox balance and the accumulation of ROS can have significant implications for cellular function and contribute to the development or progression of age-related conditions, including arthritis [44; 45; 46].

### Study strengths and limitations

Our study adds to the existing body of knowledge by providing further evidence supporting the positive correlation between the TyG index and arthritis. By utilizing the NHANES database, we were able to analyze a large representative sample, enhancing the generalizability of our findings. Moreover, our study contributes to the understanding of the TyG index as a potential marker for identifying individuals at risk of developing arthritis, emphasizing the relevance of metabolic health and insulin resistance in the pathogenesis of arthritis.

Nevertheless, this study has some limitations that should be acknowledged. First, the NHANES dataset used in this study is cross-sectional in nature, lacking longitudinal follow-up data. Second, although we adjusted for several relevant confounding factors, the presence of unmeasured or residual confounding cannot be entirely ruled out. Third, data on medications that could influence the TyG index, such as statins, fibrates, and antidiabetic medication, were not included. Furthermore, we did not include data on markers of inflammation that could potentially impact arthritis, such as C-reactive protein and erythrocyte sedimentation rate. In addition, the source of the diagnosis of arthritis in this study was patient self-report, and due to the existence of missing data, we did not further analyze the subtype of arthritis. Therefore, it is essential to interpret our findings with caution and consider them as preliminary evidence that warrants further investigation. Further studies are warranted to clarify the underlying mechanisms driving the observed correlation between the TyG index and arthritis. Prospective studies with longitudinal follow-up can help establish temporal relationships and better understand whether the TyG index is an independent risk factor for arthritis or merely a marker of metabolic dysfunction. Additionally, investigations into specific arthritis subtypes, such as rheumatoid arthritis or osteoarthritis, can shed light on potential variations. As a whole, our research offers insightful information about the association between the TyG index and arthritis, contributing to the cumulative knowledge in this field.

## Conclusion

The results of this research point to a relationship between elevated levels of the TyG index and a higher prevalence of arthritis. Our findings contribute to the expanding body of evidence supporting the clinical applicability of the TyG index as a predictive tool for arthritis, providing valuable insights for risk stratification and early intervention strategies in susceptible populations.

### Electronic supplementary material

Below is the link to the electronic supplementary material.

Supplementary Material 1

## Data Availability

Data are available upon request to the corresponding author.

## Abbreviations

BMI: Body mass index
CI: Confidence interval
ED: Erectile dysfunction
HOMA-IR: Homeostatic Model Assessment of Insulin Resistance
IL: Interleukin
IRS: Insulin Receptor Substrate
NHANES: National Health and Nutrition Examination Survey
OR: Odds Ratio
PIR: Poverty Income Ratio
PTP1B: Protein tyrosine phosphatase 1B
ROS: Reactive oxygen species
SES: Socioeconomic Status
SOCS: Suppressor Of Cytokine Signaling
TNF: Tumor necrosis factor
TyG: Triglyceride glucose
WHO: World Health Organization
