# Correction: The association between triglyceride glucose index and arthritis: a population-based study

**DOI:** 10.1186/s12944-024-02052-w

**Published:** 2024-02-27

**Authors:** Yuxin Yan, Liyu Zhou, Rui La, Ming Jiang, Dinghua Jiang, Lixin Huang, Wu Xu, Qian Wu

**Affiliations:** Department of Orthopedic Surgery, Orthopedic Institute, The First Affiliated Hospital, Suzhou Medical College, Soochow University, Suzhou, Jiangsu China

**Correction: Lipids in Health and Disease 22, 132 (2023)**

10.1186/s12944-023-01899-9

Following publication of the original article [1], the authors have identified errors in Introduction section and Table 5.

The corrections are as follows:

1. In the Introduction section, the original sentence reads: “Arthritis, a complex and widespread medical condition, is characterized by inflammation and stiffness in one or more joints of the body, with the most prevalent forms being osteoarthritis, rheumatoid arthritis, and psoriatic arthritis.” This should be corrected to: “It is characterized by inflammation and stiffness in one or more joints of the body, with the most prevalent forms being osteoarthritis, rheumatoid arthritis, and psoriatic arthritis.”
2. In Table 5, the following data need updating:

- Change 8.51 to 8.08.
- Change 1.70 (1.38, 2.09) < 0.0001 to 2.01 (1.46, 2.75) < 0.0001.
- Change 0.98 (0.86, 1.12) 0.7857 to 1.05 (0.97, 1.15) 0.2235.

The original article [1] has been updated.
